# An Overview of Light Chain Multiple Myeloma: Clinical Characteristics and Rarities, Management Strategies, and Disease Monitoring

**DOI:** 10.7759/cureus.3148

**Published:** 2018-08-15

**Authors:** Abdul Rafae, Mustafa N Malik, Muhammad Abu Zar, Seren Durer, Ceren Durer

**Affiliations:** 1 Hematology and Oncology, The University of Arizona, Tucson, USA; 2 Hematology and Oncology, University of Arizona, Tucson, USA; 3 Hemtology and Oncology, The University of Arizona, Tucson, USA

**Keywords:** light chain only multiple myeloma, bortezomib, survival

## Abstract

Light chain multiple myeloma (LCMM) constitutes approximately 15% of patients with multiple myeloma (MM). It has a poorer prognosis when compared to immunoglobulin (Ig) G or IgA variant. We performed a comprehensive literature search on LCMM and identified a total of 390 articles. After a detailed screening, six studies involving a total of 1054 LCMM patients were included. A literature review revealed bone pain and renal failure as the most common initial sign and symptoms while extramedullary disease (EMD) was acquired later during the progression of the disease. Bortezomib has shown superior efficacy in LCMM patients over nonbortezomib regimens as demonstrated by better overall response rate (95.5% vs. 60%), progression-free survival (PFS) (25% vs. 9% at two years), and overall survival (OS) (24% vs. 9% at five years). Moreover, better PFS was seen, when bortezomib was used in combination with bendamustine compared to dexamethasone (95% vs. 25% at two years). Similarly, better OS (90% at two years) was observed with bortezomib in combination with bendamustine. Monitoring of disease should include serum free light chain levels, as literature review revealed that serum assays were more sensitive in indicating the disease and predicting PFS and OS as compared to urine assays. We provide presentation patterns, clinical rarities, management strategies including their efficacy, and disease monitoring in patients with LCMM in our review paper.

## Introduction and background

Multiple myeloma (MM) constitutes for 10% of all hematological malignancies and 1% of all malignancies [1-2]. It is a malignant disease characterized by abnormal proliferation of plasma cells and monoclonal immunoglobulins or free light chains (FLC). The annual incidence of MM is 7.74 per 100,000 population while the annual number of deaths due to MM is 3.52 per 100,000 population [3-4]. More than 20,000 new cases are diagnosed in the United States every year [5]. The annual age-adjusted incidence in the United States is 4 per 100,000 population and has remained stable for years [6]. MM is more common in men as compared to women. The incidence of MM is higher in the African-American population when compared with the Caucasian population [7-8]. The clonal plasma cells in MM are primarily confined to the bone marrow and vessels [9]. Approximately 1%-2% of patients have extramedullary disease (EMD) at the time of diagnosis, while 8% of patients develop EMD later on with disease progression [7, 10]. EMD mainly involves skin, nasopharynx, larynx, upper respiratory tract, and central nervous system (CNS). Overall, more than 90% of EMDs are diagnosed in the head and neck; however, EMD constitutes less than 1% of all head and neck malignancies [11]. The CNS involvement is estimated in 1% of patients and exhibits a poor prognosis with a median survival reported to be one to two months [12-13]. The five-year overall survival (OS) and progression-free survival (PFS) among patients who initially presented with EMD at diagnosis were found to be significantly lower when compared to patients who initially presented with MM confined to bone marrow (31% vs. 59% and 21% vs. 50%) respectively [14]. The median age in patients with MM at the time of diagnosis is 65 years [7, 15]. Clinical manifestations include osteolytic bone lesions, anemia, hypercalcemia, renal insufficiency, and an increased risk of infections. Bone disease is the major cause of morbidity in patients with MM and can be detected on skeletal radiographs, magnetic resonance imaging (MRI), or computed tomographic (CT) scans [10, 16].

The most common type of M-protein found in MM is immunoglobulin (Ig) G followed by IgA [17-18]. Light chain only variant constitutes approximately 15% of patients with MM [19]. Renal failure, bone disease, and systemic light chain AL amyloidosis appear to be more frequent in patients with light chain multiple myeloma (LCMM). LCMM has an earlier average age of onset and appears to have a poorer prognosis when compared to IgG or IgA variant [3, 17]. In patients with LCMM, plasma cells show rearrangements in immunoglobulin heavy chains (IgH) at the DNA level thereby resulting in an inability to produce IgH. In most instances, one IgH allele has a germline configuration (for the D, J, and C domains), whereas the second allele is involved in a translocation. This finding is in contrast with classical MM in which one allele has a functional rearrangement, whereas the second allele is usually involved in a translocation [20].

The main aim of our analysis is to study the published literature on clinical characteristics and rarities, treatment options including their efficacy, and disease monitoring in patients with LCMM.

## Review

Methods

On 06/02/2018, a comprehensive literature search was performed on articles published after 2012 using MeSH terms, Emtree terms, and keywords on the following five databases (PubMed/Medline, Elsevier/Embase, Wiley/Cochrane Library, Thomas Reuters/Web of Science, and ClinicalTrials.gov). Search results were not limited to any geographical area. The literature search identified a total of 390 articles.

Studies fulfilling the following criteria were included as following: (1) studies investigating the efficacy and safety of bortezomib-based regimens in LCMM patient population; (2) studies having either overall response rate (ORR) or very good partial response or better (≥VGPR); (3) studies focusing on clinical presentation patterns, clinical rarities, and disease monitoring in LCMM patient population. We included only studies in the English language. If multiple publications were available for a given study, the article with the most recent publication date was included.

Two independent reviewers (AR, MNM) initially screened all retrieved titles and abstracts for relevance. The same protocol was used to screen the full text of the articles. Any conflicts were resolved with discussion. After a detailed screening, six studies involving a total of 1054 LCMM patients were included.

One author (AR) extracted the data, which were subsequently examined by the second author (MNM). We analyzed the following variables: author, year, study design, number of subjects (intervention group), median age, bortezomib regimen, dosage, ORR, complete response (CR), VGPR, partial response (PR), OS, PFS, ≥ grade 3, 4 adverse effects, sign and symptoms at initial presentation, sign and symptoms at disease progression, disease monitoring by urine protein electrophoresis (UPEP), serum free light chain (sFLC) level, and sFLC ratio. If the desired data were not reported in a study, we documented it as not specified (NS). We summarized numeric and categorical data using statistical analysis.

Results

In a study by Zhang et al. (2014) including 96 patients, initial signs and symptoms were bone pain in 74 (77.1%) patients, weakness and fatigue in 12 (12.5%) patients, renal failure in 36 (37.5%) patients, and EMD was seen in two (2.1%) patients. During the progression of disease, 78 (81.3%) patients went on to develop >3 lytic bone lesions, 26 (27.1%) patients developed pleural effusion, 32 (33.3%) patients developed EMD, 36 (37.5%) patients developed anemia, four (4.2%) patients developed hypercalcemia, one (1.04%) patient developed leukemia, and one (1.04%) patient developed M protein and skin changes (POEMS syndrome) (Table 1) [17].

**Table 1:** Clinical features observed in patients with light chain multiple myeloma. EMD, extramedullary disease; *N*, number of patients; S/S: signs and symptoms.

Author	Zhang et al. [17]
Year	2014
*N*	96
S/S at initial presentation	
S/S	*N*
Bone pain	74 (77.1%)
Renal failure	36 (37.5%)
Weakness	12 (12.5%)
EMD	2 (2.2%)
S/S developed during disease progression	
S/S	*N*
> 3 Lytic bone lesions	78 (81.3%)
EMD	32 (33.3%)
Pleural effusion	26 (27.1%)
Anemia	4 (4.2%)
Hypercalcemia	1 (1.04%)
POEMS syndrome	1 (1.04%)

In this same study, 66 patients received four cycles of bortezomib (V) (1 mg/m2) with dexamethasone (D) (20 mg) while 30 patients received nonbortezomib regimens. In bortezomib group, the ORR was 95.5%. CR was seen in 56.1% patients while 39.4% patients showed PR. In nonbortezomib group, ORR was 60%. CR was seen in 10% patients while 50% patients showed PR. The OS at three and five years in bortezomib group was 33% and 24%, respectively, while the OS in nonbortezomib group was 28% and 9%, respectively (p = 0.335). The PFS at one, two, and three years in bortezomib group was 37%, 25%, and 8%, respectively, while the PFS at one and two years in nonbortezomib group was 27% and 9%, respectively (p = 0.036). Out of 36 patients who developed renal failure, 12 (33.3%) patients with stage II disease and three (8.3%) patients with stage III recovered normal kidney function with bortezomib (Table 2) [17].

**Table 2:** Efficacy of bortezomib and nonbortezomib based regimens in treatment of light chain multiple myeloma. B, bendamustine; CR, complete response; D, dexamethasone; N.S, not specified; ORR, overall response rate; OS, overall survival; P, prednisone; PFS, progression free survival; PR, partial response; V, bortezomib; VGPR, very good partial response.

Author		Zhang et al. [17]	Zhang et al. [17]	Mrachacz et al. [21], Tessenow et al. [22]		Heaney et al. [23]
Year		2014	2014	2015, 2017		2017
Patients evaluated		66	30	45		132
Regimens		V + D	Non-V regimens	V+B+P		V
ORR		95.50%	60%	95.50%		98.40%
CR		56.10%	10%	37.70%		43.10%
VGPR		N.S.	N.S.	22.20%		44.60%
PR		39.40%	50%	35.50%		10.60%
PFS at one year		37%	27%	N.S.		N.S.
PFS at two years		25%	9%	95%		N.S.
PFS at three years		8%	N.S.	96%		N.S.
OS at three years		33%	28%	68%		N.S.
OS at five years		24%	9%	N.S.		N.S.

In two different studies by Mrachacz et al. (2015) and Tessenow et al. (2017), a total of 45 patients were treated with V (1.3 mg/m2) in combination with bendamustine (B) (60 mg/m2) and prednisone (100 mg). The ORR was 95.5% including 37.7% patients with CR, 22.2% patients with VGPR, and 35.5% patients with PR. The median OS at 24 and 30 months was 95% and 96%, respectively, while PFS at 24 and 30 months was 90% and 68%, respectively. Out of 32 patients who developed moderate to severe renal failure, 22 (68.7%) showed improvement in their renal function. The most common severe side effects were grade III-IV leukopenia in 22.2% patients and grade III-IV thrombocytopenia in 13.3% patients. Moderate to severe infection was seen in 22.2% patients (Table 2) [21-22].

In another study by Heaney et al. (2017) including 576 patients, 567 (98.4%) patients were identified by sFLC level compared to 460 (79.8%) patients who were identified by urine free light chain (uFLC) level. Three patients had unmeasurable disease by both uFLC and sFLC. One hundred thirty-two patients were evaluated, with a baseline sFLC ratio of 3207.8 mg/L which reduced to 19.7 mg/L, at a maximum response to therapy with bortezomib. The ORR was 98.4% including 43.1% patients with CR, 44.6% patients with VGPR, 10.6% patients with PR, and 1.5% patients had stable disease (SD) (Table 2). In patients who achieved CR or VGPR, significantly better PFS (p = < 0.0001; hazard ratio, HR = 0.34; 95% CI = 0.27- 0.44) and OS (p = <0.0001; HR = 0.37; 95% CI = 0.27-0.51) were observed when compared to patients with PR or SD [23].

In a study by Dejoie et al. (2016) including 113 patients, UPEP was positive in 87 (78.3%) out of 111 evaluable patients, with a median value of 1350 mg/24 h (r = 10-11,400). Serum involved free light chain (iFLC) (i.e., the light chains produced by the tumor) was positive in all (113) patients, with a median value of 1890 mg/L (r =111-274,000). Seventy-eight percent, 37%, and 18% patients showed abnormal UPEP at baseline (diagnosis), treatment cycle one and treatment cycle three, respectively, while 100%, 71%, and 46% of patients showed abnormal iFLC at baseline, treatment cycle one and treatment cycle three, respectively, indicating sFLC as the more sensitive indicator of the disease than UPEP. Abnormal iFLC level at the end of consolidation therapy showed a statistically significant shorter PFS than patients with normal iFLC level (p = 0.004; HR = 2.7; 95% CI = 1.4-5.4). UPEP did not reach any statistical significance in determining PFS (p = 0.178; HR = 1.6; 95% CI = 0.8-3.3). No statistically significant data were found for abnormal iFLC and abnormal UPEP in determining the OS [(p = 0.164; HR = 2.2; 95% CI = 0.7-6.6) and (p = 0.891; HR = 0.9; 95% CI = 0.2-4.0) respectively]. However, abnormal sFLC ratio (i.e., involved-to-uninvolved sFLC ratio) at the end of consolidation therapy showed a statistically significant shorter PFS (p = 0.006; HR = 3.1; 95% CI = 1.4-6.8) as well as OS (p = 0.047; HR = 7.8; 95% CI = 1.0-58.5) (Table 3) [24].

**Table 3:** Measure of disease and treatment response in light chain multiple myeloma patients using urine and serum assays. CI, confidence interval; iFLC, involved free light chains; *N*, number of patients; *r*, range; sFLC, serum free light chains; UPEP, urine protein electrophoresis.

Baseline parameters			
Author		Dejoie et al. [24]	
Year	2016		
N	113		
Median age	59 (*r* = 29–66)		
UPEP positive patients	78% (87/111)		
iFLC positive patients	100% (113/113)		
UPEP positive patients (%)			
Baseline		78%	
Treatment cycle 1	37%		
Treatment cycle 3	18%		
iFLC positive patients (%)			
Baseline	100%		
Treatment cycle 1	71%		
Treatment cycle 3	46%		
Progression-free survival (PFS)			
	p-value		
Abnormal UPEP	0.178 (95% CI: 0.8–3.3)		
Abnormal iFLC	0.004 (95% CI: 1.4–5.4)		
Abnormal sFLC ratio	0.006 (95% CI: 1.4–6.8)		
Overall survival (OS)			
	p-value		
Abnormal UPEP	0.89 (95% CI: 0.2–4.0)		
Abnormal iFLC	0.164 (95% CI: 0.7–6.6)		
Abnormal sFLC ratio	0.047 (95% CI: 1.0–58.5)		

In a similar study by Bradwell et al. including 224 patients, 82 patients were evaluated at follow up after chemotherapy with vincristine, doxorubicin, and high dose melphalan. CR was seen in 26 (31.7%) patients as indicated by normal uFLC levels compared with only nine (10.9%) patients by their normal sFLC levels, indicating false negative results with uFLC compared to sFLC [25].

Discussion**

Light chain multiple myeloma is the third most common type of MM and carries a grim prognosis [17]. Plasma cells in LCMM vary in their morphological presentation from a degree of maturity to degree of anaplasia [26]. On rare occasions, signet ring-like morphology is also seen, characterized by single large cytoplasmic vacuole with an eccentric flattened nucleus [27]. Auer rod-like inclusions within plasma cells, specifically in LCMM have also been reported [28].

In the patients diagnosed with LCMM, the most common signs and symptoms at disease presentation were bone pain, weakness, and renal failure. Lytic bone lesions, pleural effusion, EMD, anemia, and hypercalcemia were the complications observed as the disease progressed. One patient also developed POEMS syndrome (Table 1) [17]. These findings are similar to the one observed in other types of MM as described by Rajikumar et al. (2016) [29]. However, renal involvement is seen more commonly in LCMM as compared to other types of MM (Table 1) [3, 17].

Renal disease in MM is commonly due to circulating immunoglobulins and FLCs resulting in tubular nephropathy, known as myeloma cast nephropathy (MCN) [30]. MCN is characterized by crystalline deposition and subsequent precipitation of monoclonal FLCs either κ or λ within distal tubules [31]. Mostly, MCN occurs when serum FLC levels rise above 100 mg/dL and FLC levels less than 70 mg/dL are very rare [32]. High FLC concentrations in the proximal tubule of the kidney overwhelm the reabsorption capacity due to which FLCs pass into the loop of Henle where they bind with Tamm-Horsfall protein and subsequently results in the formation of casts in the distal tubules. Histologically, intra-tubular light chain casts with hard and fractured appearance are seen intracellularly within the distal tubules and collecting ducts. Mononuclear cells are recruited in an attempt to remove these light chain casts thereby resulting in a giant cell reaction around the casts [33]. Very rarely, FLC deposition and crystallization occurs within proximal tubules known as light chain proximal tubulopathy (LCPT) [34]. Occasionally, FLC deposition occurs in interstitial histiocytes resulting in crystal-storing histiocytosis (CSH) [35]. In LCPT and CSH, FLCs are typical of κ type and possess certain innate chemical properties that resist them against proteolytic degeneration, thereby promoting aggregation and crystallization [36]. Recently, noncrystalline morphology has been included in the histological spectrum of LCPT. Vacuoles or granules are seen in the cytoplasm of proximal tubular cells in noncrystalline morphology. From the time of renal biopsy, median renal survival is shorter for noncrystalline morphology (64 months ± 17.8) when compared to the crystalline morphology of LCPT (135 months ± 5.5) while the prognosis in case of CSH remains unclear [37]. The simultaneous occurrence of all three histological presentations (MCN, LCPT, and CSH) in MM patient is a clinical rarity [33].

A rare but very serious comorbidity of LCMM is systemic light chain AL amyloidosis. It is observed only in 5%–10% of cases of LCMM [38]. Large amounts of monoclonal light chains produced by plasma cells aggregate in tissues in the form of insoluble fibrils that form amyloid [39]. Under a light microscope using Congo red stain, homogenous red deposits are usually seen that produce apple-green birefringence under polarized light. Any organ except brain can be involved in patients with AL amyloidosis, but heart and kidney are the most frequently involved organs. Involvement of skin particularly painful sclerotic skin changes on the extremities occurs only in 25% of patients. The number of involved organs usually determines the prognosis in these patients. Involvement of more than two organs usually indicates a poor prognosis. Without treatment, the median survival is estimated to be about 13 months [38].

Other rare clinical presentations in patients with LCMM include a liver plasmacytoma presenting as a nodular lesion, jaundice, and pain in the right hypochondrium. Single or multiple space-occupying lesions, hepatomegaly, extrahepatic biliary obstruction, and ascites can be seen with an aggressive disease which is associated with a very poor outcome even with aggressive management [40]. Another rare presentation is an epidural plasmacytoid tumor in the background of LCMM presenting as shooting back pain, pathologic vertebral fracture, and weight loss [41]. Involvement of mediastinal lymph nodes in the background of dual LCMM (i.e., both lambda and kappa light chains positive cells) is also a very rare clinical presentation [14]. Subglottic plasmacytoma presenting as benign nodular lesion at the subglottis and adult-onset asthma (dyspnea and expiratory wheeze) is a clinical rarity. It is usually treated locally with CO2 laser excision and systemic therapy for treating the underlying MM. Werner in 1991 reported 111 cases of laryngeal plasmacytoma of which 21 cases had underlying LCMM [11]. Wein in 2002 reviewed 12 cases of plasmacytoma of subglottis. The average age at the time of diagnosis was 53 years, with a male to female predominance of 2:1. Six of the 12 patients had LCMM. Presenting symptoms were shortness of breath and hoarseness. Stabilization of airway via tracheostomy was needed in 58% of patients. Patients were treated mainly with localized radiotherapy [42].

Skin involvement in patients with MM is rare and occurs only in the later stage of the disease. It usually results from direct spread from an underlying osteolytic bony lesion. Lesions appear as red or violaceous, firm papules or nodules with a smooth surface ranging from 1 to 5 cm in diameter, usually on trunk and abdomen. Rarely, larger plaques like lesions are also seen [43]. Histologically, nodular or diffuse interstitial infiltration is usually seen. Literature review reveals that MM patients with cutaneous involvement show immunoglobulin (Ig) G in 56%, IgA in 24%, FLCs in 12%, IgD in 4%, and IgM in 4% of patients [44]. Bayer-Garner et al. in his study of 284 MM patients found that only 14 patients had skin lesions at the time of diagnosis. Out of these 14 patients, 10 patients were IgG variants (4 λ, 6 κ), one patient was IgA variant (κ), one patient was IgM variant (κ), one patient was a nonsecretory variant, and one patient was κ-light chain variant [39].

Progression of LCMM into secondary plasma cell leukemia (SPCL) can occur and this progression is usually accompanied by peripheral blood eosinophilia (PBE). It has been suggested that this progression from LCMM into SPCL triggered few genetic or functional alterations leading to PBE which was not present at the time of initial diagnosis [45]. Interestingly, PBE is an indicator of bad prognosis in solid malignancies which can explain the poorer outcome with SPCL when compared to primary PCL [46]. Several hypotheses have been suggested in order to explain the mechanisms triggering eosinophilia in patients with MM. Production of eosinophils may be triggered directly by proteins released from necrotic tumor cells or growth factors produced by leukocytes during immune response against the malignant cells. The growth factors released from cytokines produced by the tumor cells themselves may also trigger eosinophilopoesis. Moreover, production of eosinophils may be due to a genetically determined familial response to malignancies [45]. Direct stimulation of eosinophils by the FLCs may be another possible explanation for eosinophilia in patients with LCMM [47]. Data from larger study populations are needed in order to establish a causative relationship between PCL and PBE and to determine the various mechanisms of increased eosinophil production in patients with plasma cell dyscrasias such as LCMM.

It has been suggested that LDH level is an independent prognostic factor in patients with MM and can be associated with drug resistance. High lactate dehydrogenase (LDH) levels are also associated with EMD. Elevated LDH level in a patient with MM should alert the clinician to the possibility of EMD, but significant data are lacking [14, 44].

Bortezomib and bendamustine are FDA-approved drugs for the treatment of relapsed refractory MM and chronic lymphoid leukemia, respectively, and they have shown excellent results when studied in the patients with LCMM [17, 48]. In the study by Zhang et al. (2014), patients treated with bortezomib (V) in combination with dexamethasone (D) showed an ORR of >95% compared to an ORR of 60% in patients treated with nonbortezomib regimens (Table 2) [17]. A statistically significant better PFS was seen in the bortezomib group compared to nonbortezomib group (25% vs. 9% at two years). Patients in the bortezomib group also showed better OS compared to nonbortezomib group (24% vs. 9% at five years), but a statistically significant difference was not found.

In the study by Mrachacz et al. (2015) and Tessenow et al. (2017) patients treated with a combination of bortezomib, bendamustine (B), and prednisone (P) showed an ORR of > 95% (Table 2) [21-22]. However, better PFS was seen, when bortezomib was used in combination with bendamustine compared to dexamethasone (95% vs. 25% at two years). Similarly, better OS (90% at two years) was observed with bortezomib in combination with bendamustine. Leukopenia, thrombocytopenia, and moderate infections were the side effects observed in a few patients. Moreover, more patients showed improvement in their renal function with B+P+V compared to V+D (68.7% vs. 41.6%).

According to the International Myeloma Working Group (IMWG) guidelines, UPEP and serum iFLC are the measures used to monitor patients with LCMM [49]. Dejoie et al. (2016) in his study demonstrated various tools used for diagnosing and monitoring of patients with LCMM (Table 3) [24]. The study showed that at the time of diagnosis, serum iFLC was a more sensitive measure of the disease compared to UPEP as demonstrated by 100% detection rate by the former. Similar results were demonstrated during monitoring of the disease at treatment cycle one and three as more patients were detected by serum iFLC compared to UPEP. In the assessment of urine FLC (uFLC) and serum iFLC at treatment cycle one and three, uFLC levels showed a greater degree of response as they became negative in more patients while the serum iFLC levels were still abnormal in most of these patients. This result showed that urine samples underestimate the production of FLC and thereby falsely predict the treatment response because FLC are reabsorbed and metabolized by the kidneys, and their level might be affected by the level of kidney function.

Furthermore, an abnormal serum iFLC level at the end of consolidation therapy showed a statistically significant shorter PFS than patients with normal iFLC levels. However, no statistically significant association of serum iFLC with OS was found [24]. The sFLC ratio was found even more prognostic than serum iFLC as it was significantly predictive of PFS as well as OS. UPEP did not reach any statistical significance in determining PFS or OS. A similar finding of serum iFLC as a sensitive indicator of disease in LCMM patients was found in a study by Bradwell et al. (2003), in which the serum iFLC level remained abnormal in most of the patients after treatment, where uFLC level became normal otherwise [25]. Haeney et al. (2017) demonstrated similar results, as more patients were detected by serum iFLC compared to uFLC [23]. 

## Conclusions

There is a paucity of data on LCMM in the literature. In our study, we report that the most common presentations at the time of diagnosis were bone pain and renal failure while lytic bone lesions and EMD were acquired later in the course of the disease. Bortezomib has shown superior efficacy in LCMM patients over nonbortezomib regimens with an ORR > 95%. Moreover, bortezomib in combination with bendamustine has shown better PFS and OS compared to bortezomib in combination with dexamethasone. Moreover, sFLC levels were more sensitive in indicating the disease and predicting PFS and OS as compared to uFLC levels. Hence monitoring of LCMM patients should include serum assays. Recently, serum N-glucan has been introduced as a potential biomarker for routine diagnosis and monitoring of LCMM patients. However, significant data are lacking, and data from larger study populations are needed.
